# Deciphering the Niches of Colonisation of *Vitis vinifera* L. by the Esca-Associated Fungus *Phaeoacremonium aleophilum* Using a *gfp* Marked Strain and Cutting Systems

**DOI:** 10.1371/journal.pone.0126851

**Published:** 2015-06-10

**Authors:** Romain Pierron, Markus Gorfer, Harald Berger, Alban Jacques, Angela Sessitsch, Joseph Strauss, Stéphane Compant

**Affiliations:** 1 Université de Toulouse, Equipe Vins Viticulture et Œnologie, Département des Sciences Agronomiques et Agroalimentaires, INP-EI Purpan, 75 voie du T.O.E.C. BP57611, Toulouse, France; 2 University of Natural Resources and Life Sciences, Department of Applied Genetics and Cell Biology, Tulln, Austria; 3 AIT Austrian Institute of Technology GmbH, Health & Environment Department, Bioresources Unit, Tulln, Austria; University of Milan, ITALY

## Abstract

**Introduction:**

Esca disease has become a major threat for viticulture. *Phaeoacremonium aleophilum* is considered a pioneer of the esca complex pathosystem, but its colonisation behaviour inside plants remains poorly investigated.

**Material and Methods:**

In this study, *P*. *aleophilum*::*gfp7* colonisation was assessed six and twelve weeks post-inoculation in two different types of tissues: in the node and the internode of one year-old rooted cuttings of Cabernet Sauvignon. These processes of colonisation were compared with the colonisation by the wild-type strain using a non-specific lectin probe Alexa Fluor 488-WGA.

**Results:**

Data showed that six weeks post-inoculation of the internode, the fungus had colonised the inoculation point, the bark and xylem fibres. Bark, pith and xylem fibres were strongly colonised by the fungus twelve weeks post-inoculation and it can progress up to 8 mm from the point of inoculation using pith, bark and fibres. *P*. *aleophilum* was additionally detected in the lumen of xylem vessels in which tyloses blocked its progression. Different plant responses in specific tissues were additionally visualised. Inoculation of nodes led to restricted colonisation of *P*. *aleophilum* and this colonisation was associated with a plant response six weeks post-inoculation. The fungus was however detected in xylem vessels, bark and inside the pith twelve weeks post-inoculation.

**Conclusions:**

These results demonstrate that *P*. *aleophilum* colonisation can vary according to the type of tissues and the type of spread using pith, bark and fibres. Woody tissues can respond to the injury and to the presence of this fungus, and xylem fibres play a key role in the early colonisation of the internode by *P*. *aleophilum* before the fungus can colonise xylem vessels.

## Introduction

During the last twenty years, grapevine trunk diseases (GTDs) in general and esca in particular have become one of the major concerns in viticulture [1,2]. GTDs affect 12% of the French vineyard, suffering from different pathologies such as Eutypa dieback, Botryosphaeria dieback or esca disease. Esca has become particularly important for winegrowers since the banning of sodium arsenite due to its toxicity towards humans and the environment [3]. This disease causes significant losses of production and imposes severe threats by increasing plant renewal rate in mature vineyards [4]. No current solution can prevent this disease, although esca is probably known since ancient Greek times [5]. It is characterised by a brown red discoloration and black streaking in the xylem (considered as ‘young esca’) with the later development of a white rot in the trunk (‘esca proper’). Both young esca and esca proper in the trunk are associated with leaf tiger stripe symptoms as well as symptoms on berries [6–8]. Plants with heavily damaged trunks may suddenly die due to esca symptoms. The etiology of this disease is not yet well understood [1] but the inoculum source of fungi related to the disease may be soil, air dispersal of spores, insect vectors and/or contamination during grafting processes throughout roots, pruning or natural wound colonisation [9,10]. The original inoculum may also be present in the cuttings from already infected mother vines [11].

A cocktail of microbes can be responsible for the full development of esca trunk disease [1]. Albeit *Phaeomoniella chlamydospora* (W. Gams, Crous, M.J. Wingf. & L. Mugnai) Crous & W. Gams [12] and *Phaeoacremonium aleophilum* W. Gams, Crous, M.J. Wingf. & L. Mugnai [13] are the principal tracheiphilous hyphomycetes associated with black streaking and brown-red wood [6,7], other fungi have been associated with esca symptoms in Europe, notably the wood-rotting basidiomycete *Fomitiporia mediterranea* Fischer, and occasionally *Stereum hirsutum* (Willd.: Fr) S. F. Gray [7]. Additional fungi, i.e. *Eutypa lata*, and *Botryosphaeriaceae* species, as well as several *Phaeoacremonium* spp., have been isolated from trunks of plants showing esca disease [7,14], as several diseases may occur in the same plant and fungi associated with these diseases may also be present at the same time as esca. However, *P*. *aleophilum* has been considered as a pioneer in esca disease infection, because this fungal agent is frequently isolated from trunks of plants presenting ‘young esca’. This microbe was isolated from plant tissues when decay development started in the trunk together with *P*. *chlamydospora*. Consequently *P*. *aleophilum* is an interesting model microorganism to study grapevine-microbe interaction as well as for the understanding of esca development. A better knowledge of the niches colonised by this fungus inside plants is however required.

The necessity of injury for infection by *P*. *aleophilum* has been investigated on single-bud cuttings of cv. Cabernet Sauvignon [15] using scanning electron microscopy. The pathogen was able to penetrate uninjured roots and shoots without passing by stomata, and colonised its host plant especially in the intercellular spaces of the epidermis, as well as inside the cortex and the pith. Both phloem and xylem vessels were also colonised, with the latter more extensively [15]. Isolation from dormant cuttings buried in conidia-inoculated sand revealed that *P*. *aleophilum* is able to invade cv. ‘Chardonnay’ more successfully than *P*. *chlamydospora*. This study stressed the importance of the node in the infection cycle of *Phaeoacremonium* species [16]. Electron microscopy analysis revealed that *P*. *aleophilum* is a vascular pathogen colonising wood fibres, xylem vessels as well as pith [17]. Using a FITC-WGA assay, Fleurat-Lessard *et al*. [18] visualised *P*. *aleophilum* one year after inoculation in several parts of the trunk of infected cuttings of cv. Ugni blanc, mainly in xylem vessels and fibres, but also inside proto-xylems, pith and rays.

Grapevine colonisation by *P*. *chlamydospora* is better documented than for *P*. *aleophilum* [17,19] and Troccoli *et al*. [20] described the use of different staining techniques to observe grapevine trunk pathogens *in planta*. In addition to immunological detection developed by Fleurat-Lessard *et al*. [18], as well as other techniques enabling the detection of fungi in general, marker genes (e.g. *gfp*) may be used to study the colonisation of grapevine trunk disease-associated fungi [21–24]. There is not yet, however, a study concerning the colonisation of *P*. *aleophilum* using a *gfp* marker, but such a study would enable a better understanding of the niches colonised by *P*. *aleophilum*.

In this study *gfp* tagged transformants of *P*. *aleophilum* were created using *Agrobacterium*-mediated transformation. The process of colonisation was then studied on cuttings of Cabernet Sauvignon plants after two types of inoculation: first in the internode of one year-old woody tissues already lignified, second in the node of a newly developed branch after pruning. Colonisation behaviour of *P*. *aleophilum* was further determined using the wild-type fungus with Alexa Fluor 488-WGA to elucidate which niches can be colonised by the phytopathogenic fungus.

## Material and Methods

### Fungal strains and preparation for transformation

*Phaeoacremonium aleophilum* CBS 100398 was maintained on potato dextrose agar medium (PDA, Merck, Germany) on petri dishes placed in the dark at 26°C. Prior to transformation, a test was performed to determine whether this strain was sensitive to different concentrations of hygromycine B (25 μg.mL^-1^, 50 μg.mL^-1^, 75 μg.mL^-1^ and 100 μg.mL^-1^) in PDA medium.

The transformation protocol required a conidial suspension of *P*. *aleophilum*. A plug of hyphae from a 3 week-old culture was placed in 1 mL of autoclaved demineralised water (121°C, 15min) in a 1.5 mL tube to make a conidia solution. The tube was then briefly vortexed and centrifuged for 30 s at 2300 x g. The plug of hyphae was then removed and the tube centrifuged again for 30 s at 2300 x g to allow precipitation of fungal conidia at the bottom of the tube. The conidial suspension was then concentrated by pipetting the upper part of the solution to obtain a final volume of 200 μL and the concentration was adjusted with a counting chamber (Malassez cell) to obtain 10^4^ to 10^5^ conidia.μL^-1^.

### Fungal transformation

*Agrobacterium*-mediated transformation of *P*. *aleophilum* CBS 100398 with plasmid pCBCT was carried out on young, growing hyphae from a fresh *P*. *aleophilum* conidial suspension according to Gorfer *et al*. [25]. The binary vector pCBCT contains *gfp* under the control of the strong *toxA* promoter [26] and the hygromycine resistance marker *hph*. Briefly, 50 μL of the *P*. *aleophilum* solution were sprayed on an autoclaved cellophane layer that was previously placed on PDA medium in a Petri dish. Fungal hyphae developed for one week before transferring the cellophane on a Moser-Induction medium (containing 0.2% glucose, 10 mM MES, 2 g.L^-1^ peptone, 0.2 g.L^-1^ yeast extract, 0.5 g.L^-1^ KH_2_PO_4_, 0.05 g.L^-1^ myo-inositol, 75 mg.L^-1^ CaCl_2_.2 H_2_O, 150 mg.L^-1^ MgSO_4_.7 H_2_O, 10 mg.L^-1^ MnSO_4_.H_2_O, 1 mg.L^-1^ ZnSO_4_.7H_2_O, 2% agar and 200μM acetosyringone (AS) + 0.5% glycerol added after autoclaving; pH 5.3). *A*. *tumefaciens* AGL-1 [pCBCT] was in parallel cultivated overnight on a shaker (180 rpm) at 30°C in LB medium amended with 1% glucose and kanamycin (50μg.mL^-1^). Bacterial cells were then centrifuged (3000 x g; 10 min) and the pellet was re-suspended in 2 mL of an *Agrobacterium*-induction medium (containing 10.5 g.L^-1^ K_2_HPO_4_, 4.5 g.L^-1^ KH_2_PO_4_, 1 g.L^-1^ (NH_4_)_2_SO_4_, 0.5 g.L^-1^ sodium citrate, 0.2% glucose, 8 mM MgSO_4_, 1 mg.L^-1^ thiamine, 200μM AS, 40 mM MES; pH 5.3) and incubated for 6 h at 30°C on a shaker (180 rpm).

For co-cultivation, 170 μL of induced AGL-1 [pCBCT] were spread with a Drigalsky spatula on the cellophane where *P*. *aleophilum* CBS 100398 had been growing for five days. Plates were then incubated at room temperature for five days. The cellophane was transferred onto a Moser selective medium (with 1% glucose, 2 g.L^-1^ peptone, 0.2 g.L^-1^ yeast extract, 0.5 g.L^-1^ KH_2_PO_4_, 0.05 g.L^-1^ myo-inositol, 75 mg.L^-1^ CaCl_2_.2 H_2_O, 150 mg.L^-1^ MgSO_4_.7 H_2_O, 10 mg.L^-1^ MnSO_4_.H_2_O, 1 mg.L^-1^ ZnSO_4_.7H_2_O, 50 μg.mL^-1^ HygB, 100 μg.mL^-1^ Cef, 2% Agar, pH 6.0) to isolate fungal transformants.

Transformants were transferred to a fresh selection medium. After two selection rounds, a conidia solution was sprayed on a new plate containing selection medium. This selection from conidia solution avoids the presence of heterokaryons. Hyphae developing from conidia were transferred once more on selection medium and maintained then on PDA medium. A few hyphae were mounted in sterile water with a cover slide and compared to wild-type *P*. *aleophilum* and observed under a confocal microscope (described in another section).

### Plant material

**Fig 1:**
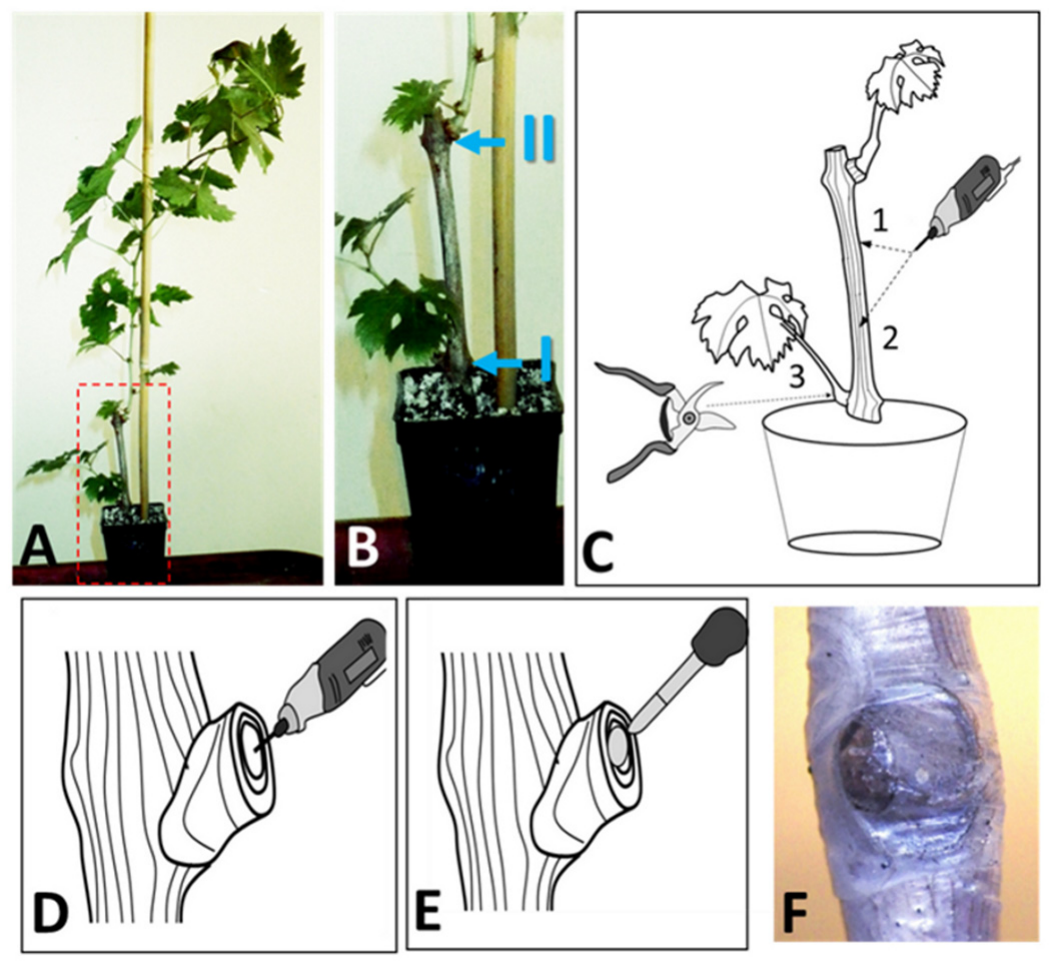
The inoculation model. A) A three noded cutting of Cabernet Sauvignon clone 15 is made so that one node is inside the soil and the two other nodes give shoots. B) Two young branches will form annotated branch I and branch II. C) Two internodal damages are realised with a driller (1 and 2). A pruning damage is done at the nodal level by cutting the branch I. D) A small wounding is made in the pruning injuries to receive the plug of the fungus to be inoculated. E) A plug of peripheral hyphae is inoculated in the internodal or nodal damage. F) The inoculation is covered with cellophane.

One year-old canes of *Vitis vinifera* L. cv. Cabernet Sauvignon clone 15 were harvested in January 2013 and 2014 (Toulouse, Midi-Pyrénées, France) and treated with fungicide by soaking canes in 0.05% Cryptonol for one hour. Cleaned canes were stored at 4°C until further processing. Canes were divided into cuttings harbouring three dormant buds and cleaned in a 20 L water bath containing 10 mL of bleach (2.5% active chloride) for 1 min before rinsing two times with tap water. Cuttings were then stored at 4°C overnight in a solution of 0.05% Cryptonol. Plant material was cleaned with three successive washes in baths of sterile tap water and planted in plastic trays filled with moistened autoclaved glass-wool. Plants were placed in a growing chamber (photoperiod 16/8, 25°C; 90% humidity) and were watered with autoclaved tap water. Budding and rooting took four to six weeks before cuttings were potted in 75 cL pots containing a sterile mixture of perlite, sand and turf (1:1:1 v/v). Cuttings were then transferred to a growth chamber (photoperiod 16/8, 25°C; 45% humidity) and plants remained there for at least one week before treatments to avoid potting stress (cf. Fig 1A).

### Plant inoculation

Cuttings (n = 70) were inoculated when at least six leaves were fully developed. Firstly, cuttings were partly surface-sterilised with a tissue sprayed with 70% ethanol. For each sampling time (6 and 12 weeks) plants were inoculated with hyphae of *P*. *aleophilum*::*gfp*7 (n = 10) from three PDA plates or with the wild-type strain of *P*. *aleophilum* (n = 10) grown on PDA. Mock-treated plants (n = 10) were inoculated with sterile PDA medium. Three inoculations were performed on each plant—two in the internode and one at nodal level as outlined below (Fig 1). Inoculations were performed with one syringe per treatment to ensure that the same quantity of hyphae was injected.

#### Internodal inoculation

Wounding damage at the internode was made using a drilling machine with a 3 mm drill head (Fig 1C). Inoculations in the same internode were separated by 3 cm along the cane and by a 90° angle (Fig 1C). A cylindrical plug (3 mm long and 1 mm diameter) of *P*. *aleophilum*::*gfp*7 growing on PDA medium was applied to the wound. The wild-type *P*. *aleophilum* CBS 100398 or control medium were applied in the same way. Only hyphae in the periphery of the growing fungus were collected to avoid the danger of selecting fungal material at a different reproductive stage or with different cell activity at different locations on the same plate. After inoculation, the wound was covered with cellophane (Fig 1).

#### Node inoculation

For node damage, the branch formed from the middle node ("I" in Fig 1B) was cut with an ethanol-flamed blade (Fig 1C). Then a wound was made at this nodal level with an ethanol-flamed 3 mm drill (Fig 1D). A plug of peripheral hyphae of the wild-type strain of *P*. *aleophilum* or *P*. *aleophilum*::*gfp*7 growing on PDA was transferred to the wounding damage as described before (Fig 1E) and control plants were inoculated with PDA medium. Inoculated injuries were covered with cellophane (Fig 1F).

Following inoculation at both internodal and node levels, plants were maintained in the growing chamber, under the same conditions, and watered every second day with autoclaved tap water. Plants were then harvested six or twelve weeks post-inoculation for microscopy.

### Plant sampling and preparation for confocal laser scanning microscopy

At sampling for microscopy, the trunks of cuttings were harvested by using sterile secateurs, collected in sterile tubes and stored at 4°C. Plants inoculated with *P*. *aleophilum*::*gfp*7 or mock-treated plants were cut longitudinally or transversely with secateurs close to the inoculation site. These wood sections (10x10x0.5 mm) were stored at 4°C (*P*. *aleophilum* does not grow at 4°C; see [17,27]) before observation under a confocal microscope. No chemical fixation was carried out on samples to avoid reduction of the GFP signal.

Similar preparation was done for wild-type *P*. *aleophilum* CBS 100398 or an additional set of mock treated plants, except that the cuttings, after longitudinal or transversal sections, were immersed in 15 ml of phosphate buffer saline (PBS, pH 7.2) containing 50 μg ml^-1^ of wheat germ agglutinin (WGA)-AlexaFluor488 conjugate (Life Technologies, USA) and incubated 2 hours at 28°C before rinsing three times with PBS and observed under the confocal microscope. WGA preferentially binds to the chitin of fungi. This staining is universal to fungi and thus all species present in the plant, *P*. *aleophilum* included, can be marked with this WGA-AlexaFluor488 conjugate. Additional microbes such as gram-positive bacteria inhabiting plant tissues can be however visualised using this technique as WGA can bind to sialic acid.

Similar preparation was carried out for samples at six or twelve weeks post-inoculation. All the data result from observations of ten cuttings of each treatment (wild-type strain of *P*. *aleophilum*, *P*. *aleophilum*::*gfp*7 and mock at six weeks post-inoculation and the same numbers twelve weeks post-inoculation).

### Confocal laser scanning microscopy

All observations of pure hyphae or treated plants were carried out using a confocal microscope (Olympus Fluoview FV1000 with multi-line laser FV5-LAMAR-2 and HeNe(G)laser FV10-LAHEG230-2, Japan). No treatments were applied to the tissues prior to observation to avoid destruction or reduction of the GFP signal from inoculated plants or for pure hyphae of the wild-type strain of *P*. *aleophilum* or *P*. *aleophilum*::*gfp*7. Similar experiments were done for the WGA-AlexaFluor488 assay.

Observations with the confocal microscope were done at objectives of 10x, 20x and 40x and between 20 and 40 X, Y, Z pictures containing 20 to 60 scans were separately taken at 405, 488, 549 nm wavelengths in blue/green/orange-red channels respectively, with the same settings each time. Imaris software was used at the confocal microscope to visualise 3D reconstructions. X, Y, Z pictures from different channels were then merged (RGB for red, green and blue merging) using the image J software 1.47v, and Z project stacks were then used to create the pictures (as described in [28]).

## Results

### CSLM microscopy from a pure culture of *P*. *aleophilum*::*gfp*7

**Fig 2:**
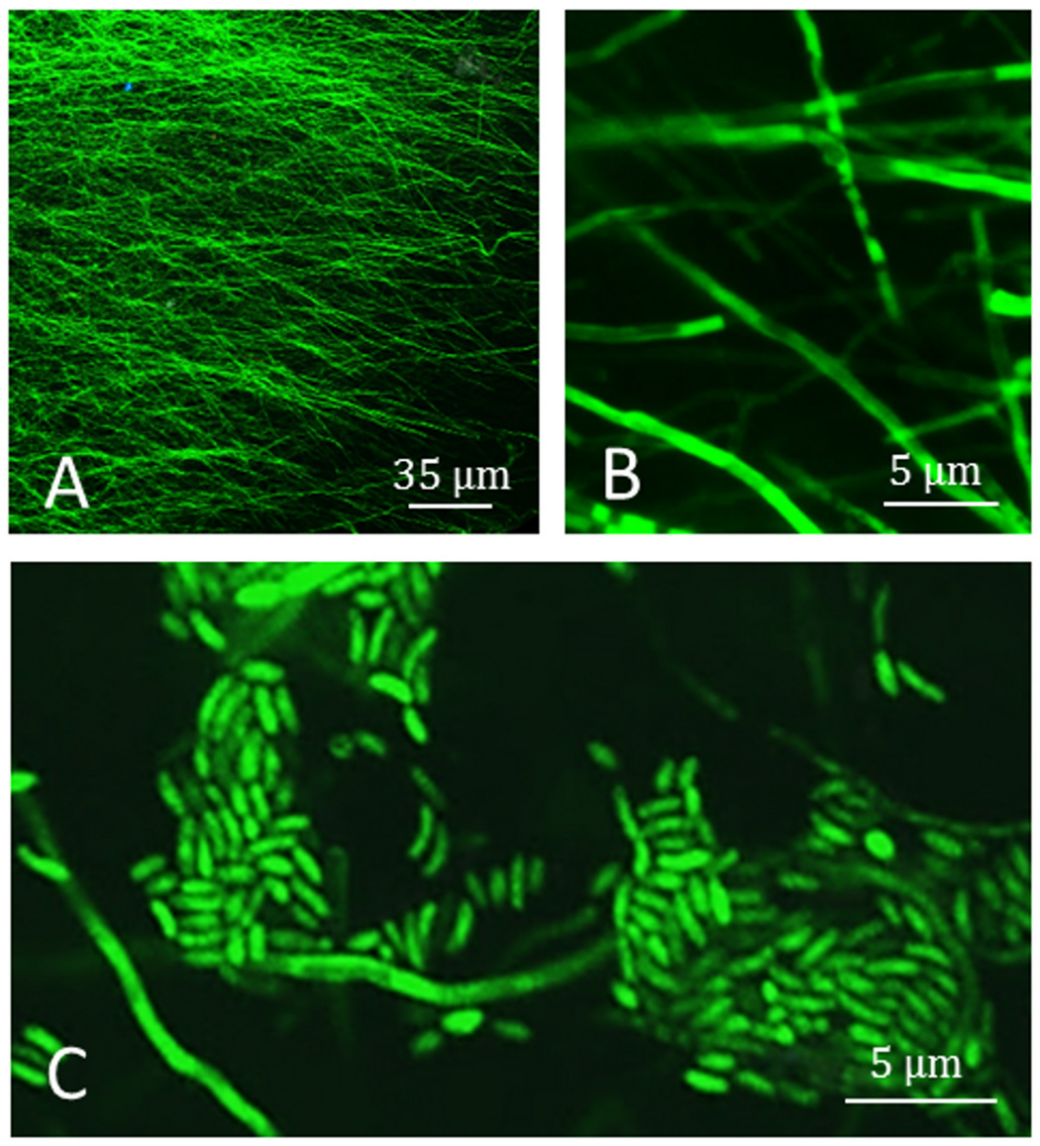
CSLM observation of *Phaeoacremonium aleophilum*::*gfp*7 from a pure culture. A) Peripheral hyphae from plate showing strong GFP fluorescence. B) Magnification of the hyphae and hyphae with punctuated fluorescence can be occasionally seen. C) Both hyphae and conidia are green fluorescent.

*P*. *aleophilum* CBS 100398 was successfully transformed with the *gfp* gene. Among seven transformed strains, strain n°7 (*P*. *aleophilum*::*gfp*7) was selected for further experiments as it presented an intense green fluorescence when exposed to UV light (Fig 2A–2C). The GFP signal was intense and continuous all over the hyphae, although occasionally punctuated distribution of the GFP signal in hyphae could be observed (see Fig 2B). Conidia were mostly aggregated and also marked with the GFP signal (Fig 2C). The wild-type strain did not present any autofluorescence that could lead to a background signal. No differences in growth between the *gfp* transformant and the wild-type were observed after fungi were grown on PDA medium (data not shown).

### CSLM microscopy of *P*. *aleophilum*::*gfp*7 six weeks post-inoculation

**Fig 3:**
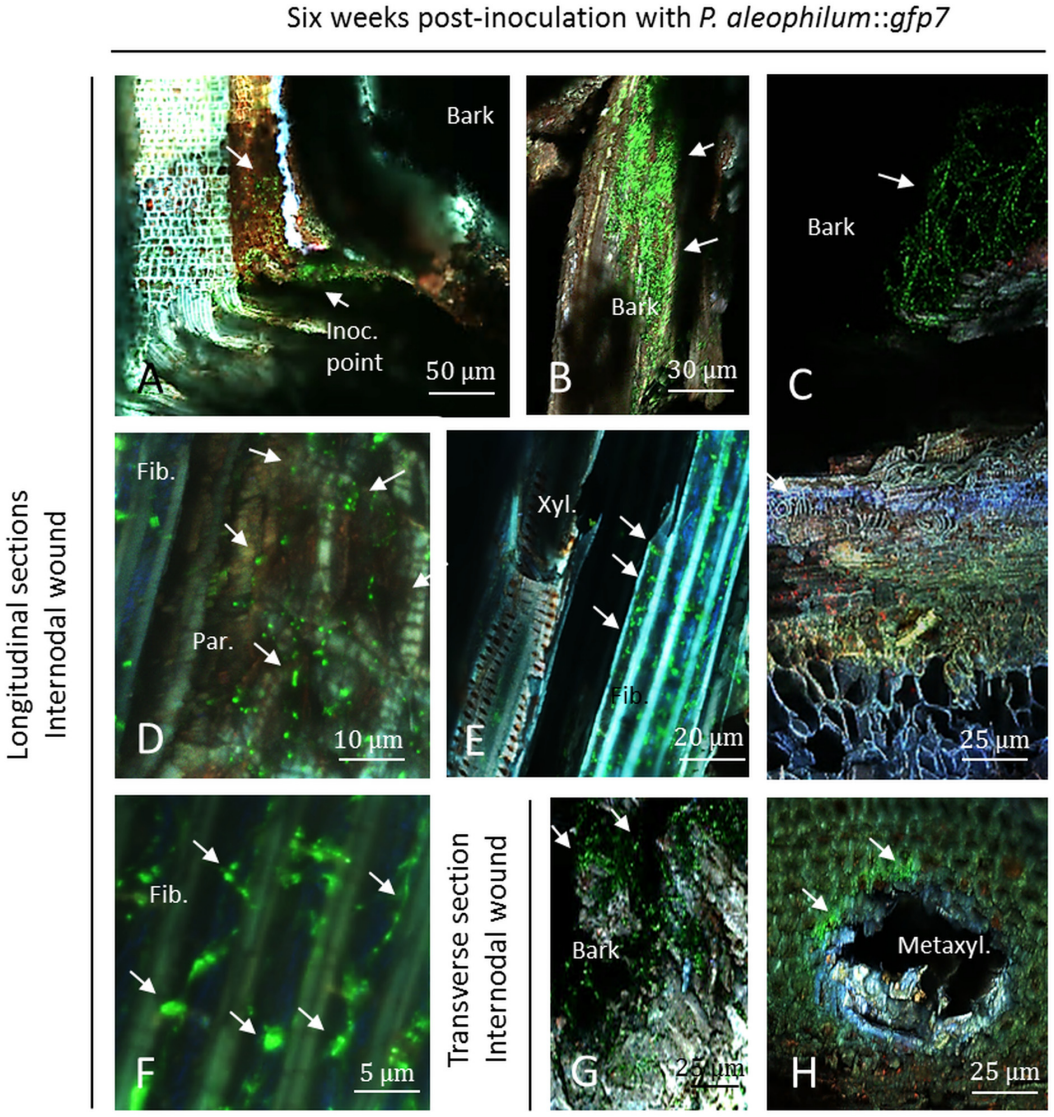
Observation of internodal longitudinal (A–F) and transverse (G–H) sections of Cabernet Sauvignon clone 15 cuttings challenged with *P*. *aleophilum*::*gfp*7 (arrows) six weeks post-inoculation. A) Inoculation point colonised by *P*. *aleophilum*::*gfp*7. B) Strong presence of hyphae in the bark surrounding the inoculation point. C) Damaged bark covered with *P*. *aleophilum*::*gfp*7 hyphae next to wood tissues of the grapevine trunk. D) Xylem parenchymal cells colonised by *P*. *aleophilum*::*gfp*7, close to xylem fibres. E–F) Hyphae and conidia are localised in xylem fibres, while scarce colonisation of xylem vessel elements are reported. G–H) Transverse section showing the localisation of *P*. *aleophilum*::*gfp*7 in the bark and in xylem fibres surrounding xylem vessels. Fib.: fibres, Inoc. point: inoculation point, Par.: parenchyma, Metaxyl.: metaxylem, Xyl.: xylem.

At the internode level and in transverse sections, the inoculation point was still clearly visible six weeks post-inoculation (Fig 3A). The bark and xylem vessels were damaged (Fig 3A). At low magnification hyphae of *P*. *aleophilum*::*gfp*7 were detected at the inoculation point (Fig 3A). A considerable quantity of hyphae was located in the bark around the inoculation point (Fig 3B). The bark around the injury was the tissue where most of the hyphae were detected (Fig 3B and 3C). Some *P*. *aleophilum*::*gfp*7 hyphae were observed in parenchymal cells in part of the samples (Fig 3D). The wood fibres separating xylem vessels and parenchyma cells were strongly colonised in the two mm next to the wound by short hyphae or germinated conidia (Fig 3E). Hyphae detected in Fig 3E seemed more punctuated than the ones observed from pure fungal culture (Fig 2) and short hyphae or alternatively conidia were detected. Observations at higher magnification confirmed that the pathogen was inside wood fibres, marked by a GFP signal punctuated among the hyphae (Fig 3F). Fig 3F illustrates the abundance of short hyphae in fibres that might have colonised the surrounding xylem vessel. The hyphae were surprisingly thin in comparison to hyphae developing on PDA medium (see Figs 2C and 3F). Short *P*. *aleophilum*::*gfp*7 hyphae clearly colonised the trunk along the fibres six weeks post-inoculation whereas even at high magnification the spreading from fibre to fibre remains speculative (Fig 3F). Hyphae of *P*. *aleophilum*::*gfp*7 were rarely observed in xylem vessels. One signal can be seen in the lumen of xylem vessels in Fig 3E but among all plants analysed only one colonisation of xylem vessel lumen was found six weeks post-inoculation. Hyphae of *P*. *aleophilum*::*gfp*7 were also detected at the inoculation point when observing transverse sections (Fig 3G and 3H). Transverse sections also confirmed that the bark surrounding the inoculation point was covered by hyphae and conidia (Fig 3G). This observation also confirmed the colonisation of fibres surrounding the metaxylem vessels (Fig 3H). No signal was detected in control plants inoculated with a plug of sterile PDA medium (data not shown). Inoculation of *P*. *aleophilum*::*gfp*7 in an internodal injury was successful and led to more successful colonisation of thin hyphae in xylem fibres compared to other tissues.

**Fig 4:**
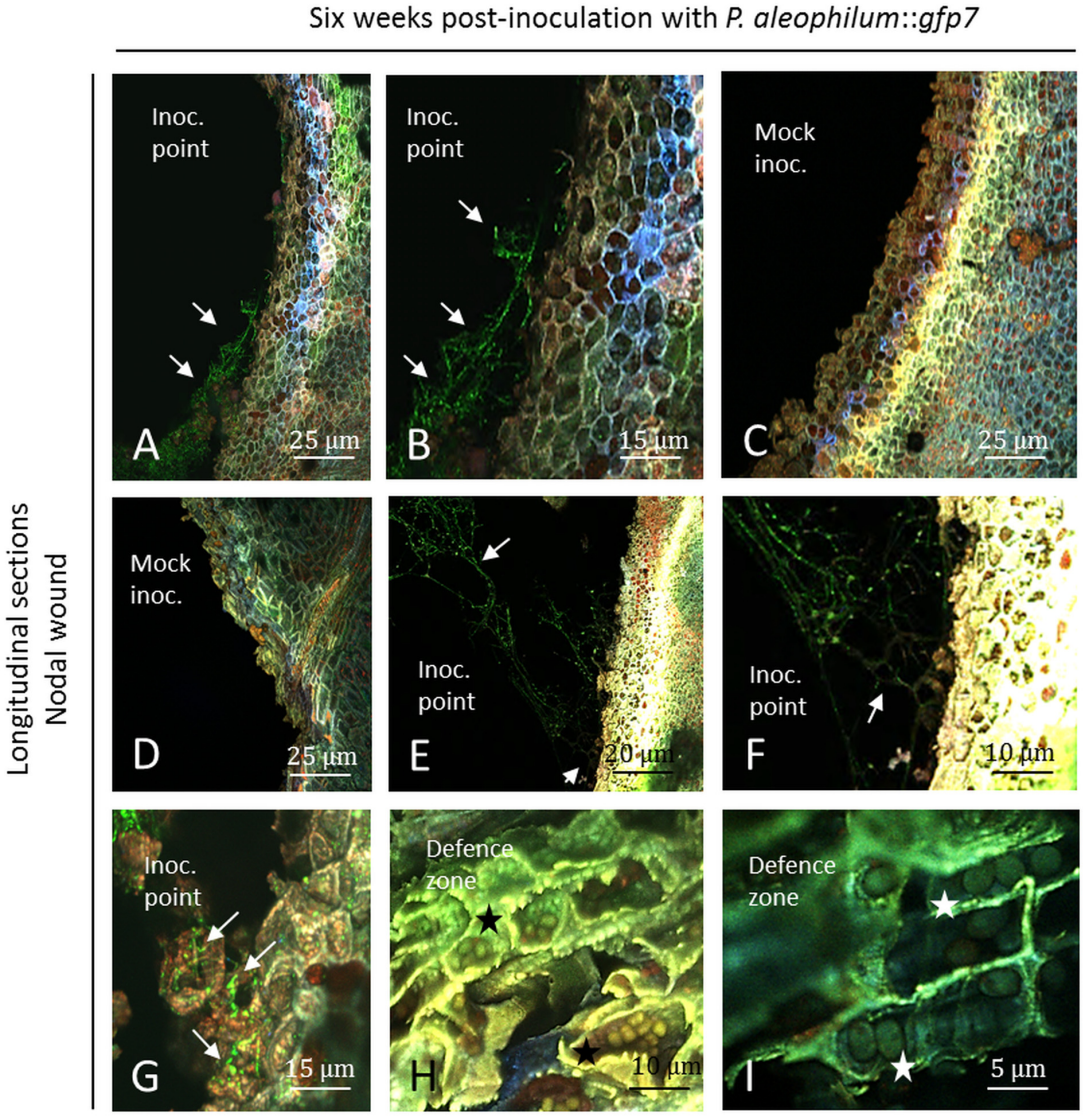
Observation of nodal transverse (A–I) sections of Cabernet Sauvignon clone 15 cuttings challenged with *P*. *aleophilum*::*gfp7* (arrows) six weeks post-inoculation. A) Inoculation point showing *P*. *aleophilum*::*gfp*7 and a strong plant response, as indicated by blue, pink and green fluorescence. Sometimes this plant defence reaction was yellow/white fluorescent. B) *P*. *aleophilum*::*gfp*7 seems to be impacted by the plant response because no hyphae was observed in front of the plant reaction zone. C–D) The wounding damage also induces plant responses in control plants ranging from clearly visible (C) to hardly visible (D), but always of lower intensity than the plant inoculated with fungal hyphae, and with blue, pink and yellow greenish fluorescence. E–F) The GFP signal disappeared from hyphae when hyphae reaching a white/yellow zone. G) A necrotic layer of cell is colonised, but the infection seems to be blocked by a yellow fluorescent zone six weeks post-inoculation. H) After the yellow fluorescent zone, a green fluorescent tissue was observed at higher magnification to ensure no fungus colonised it. I) There is no *P*. *aleophilum*::*gfp*7 in the green fluorescent cell layer, but interestingly sometimes some ovoid structures (highlighted by fluorescent cells) (asterisks) are accumulating in those cells. Inoc. point: inoculation point, Mock inoc.: mock inoculated.

Hyphae of *P*. *aleophilum*::*gfp*7 were detected at the inoculation site in damaged nodes (Fig 4A) but did not colonise the trunk any further six weeks post-inoculation. In this model, plants inoculated with the transformed fungus responded strongly by forming a zone of layers of autofluorescent cells reacting to the fungus (named hereafter reaction zone; Fig 4A and 4B). Mock-treated plants also presented a reaction zone but of a lesser intensity ranging from the one of Fig 4C and 4D. In the reaction associated with the *P*. *aleophilum*::*gfp*7 a higher fluorescence of blue, pink and green cells was observed compared to the reaction zone in the node of mock plants six weeks post-inoculation (Fig 4A, 4B, 4C and 4D). This reaction has been observed in all samples but the observed plant responses did not completely cover the inoculation point. Fig 4E shows hyphae reaching some of the injured tissues, without the same reaction zone, i.e. white, due to combination at the same place of blue, green and red fluorescing signals (due to merging RGB), but the GFP signal seemed to decrease in intensity as the hyphae approached plant tissues (Fig 4E and 4F). At the contact of this responding plant tissue, the GFP signal totally disappeared with a brown coloration of the hyphae appearing (Fig 4E and 4F). A layer of necrotic cells was observed between the inoculation point and the reaction zone (Fig 4G). This zone, next to the inoculation point, was colonised by *P*. *aleophilum* six weeks post-inoculation. Interestingly, this cell zone was filled in some samples with multiple ovoid granules, which were not observed in control plants (Fig 4H and 4I).

### CSLM visualisation of wild-type *P*. *aleophilum* CBS 100398 six weeks post-inoculation using Alexa Fluor 488-WGA

**Fig 5:**
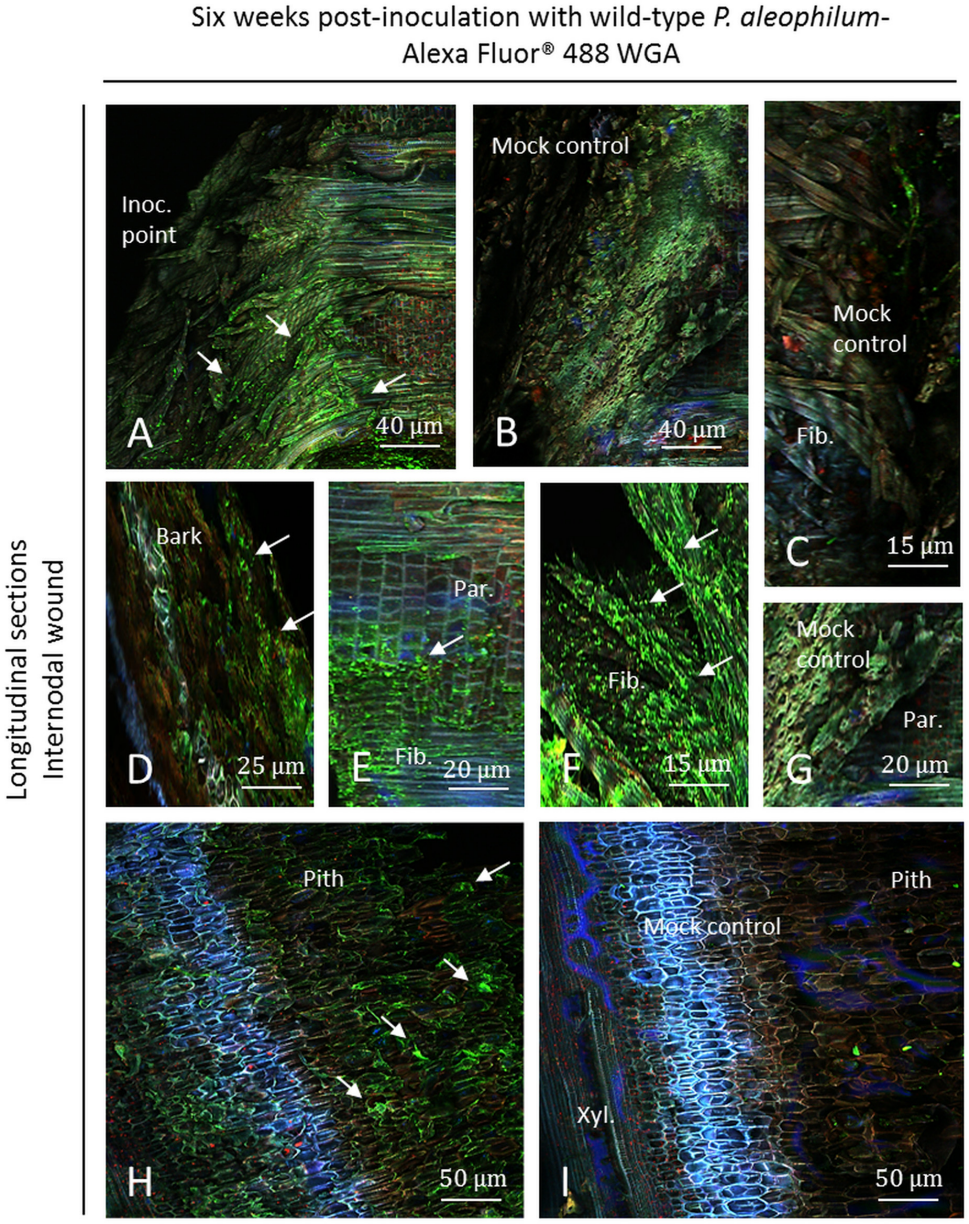
Observation of internodal longitudinal sections of Cabernet Sauvignon clone 15 cuttings challenged with wild-type *P*. *aleophilum* CBS 100398 (arrows) six weeks post-inoculation and stained with Alexa Fluor 488-WGA. A) Inoculation point colonised by *P*. *aleophilum*. B-C) Mock control. D) Damaged bark covered with wild-type *P*. *aleophilum*. E) Parenchymal cells colonised by *P*. *aleophilum*, close to xylem fibres. F) Xylem fibres strongly colonised by wild-type *P*. *aleophilum*. G) Parenchymal cells from the mock control. H) Pith colonised by few hyphae. I) Mock control pith colonised by few natural endophytes. Fib.: fibres, Inoc. point: inoculation point, Par.: parenchyma.

Using Alexa Fluor 488-WGA, hyphae or short hyphae were detected near the inoculation point at the internodal level. Fibres were especially observed to be intensively colonised in plants subjected to the *P*. *aleophilum* wild-type strain (Fig 5A) around the point of inoculation in comparison to the mock control (Fig 5B). Hyphae, conidia or germinated conidia were additionally detected in the bark (Fig 5D), few in parenchymal cells (Fig 5E) and in fibres (Fig 5F). The pith seemed to be sparsely colonised (Fig 5H) in comparison to the mock control (Fig 5I). Few microbes, that may represent natural endophytes of cuttings, were detected in control plants in different tissues (Fig 5C and 5I). In parenchyma of control plants no microbe was detected (Fig 5G). A small amount of fungi and bacteria has been additionally detected in the lumen of xylem vessels of mock-treated plants but most of the samples did not reveal any hyphae in this cell layer (Fig 5I).

**Fig 6:**
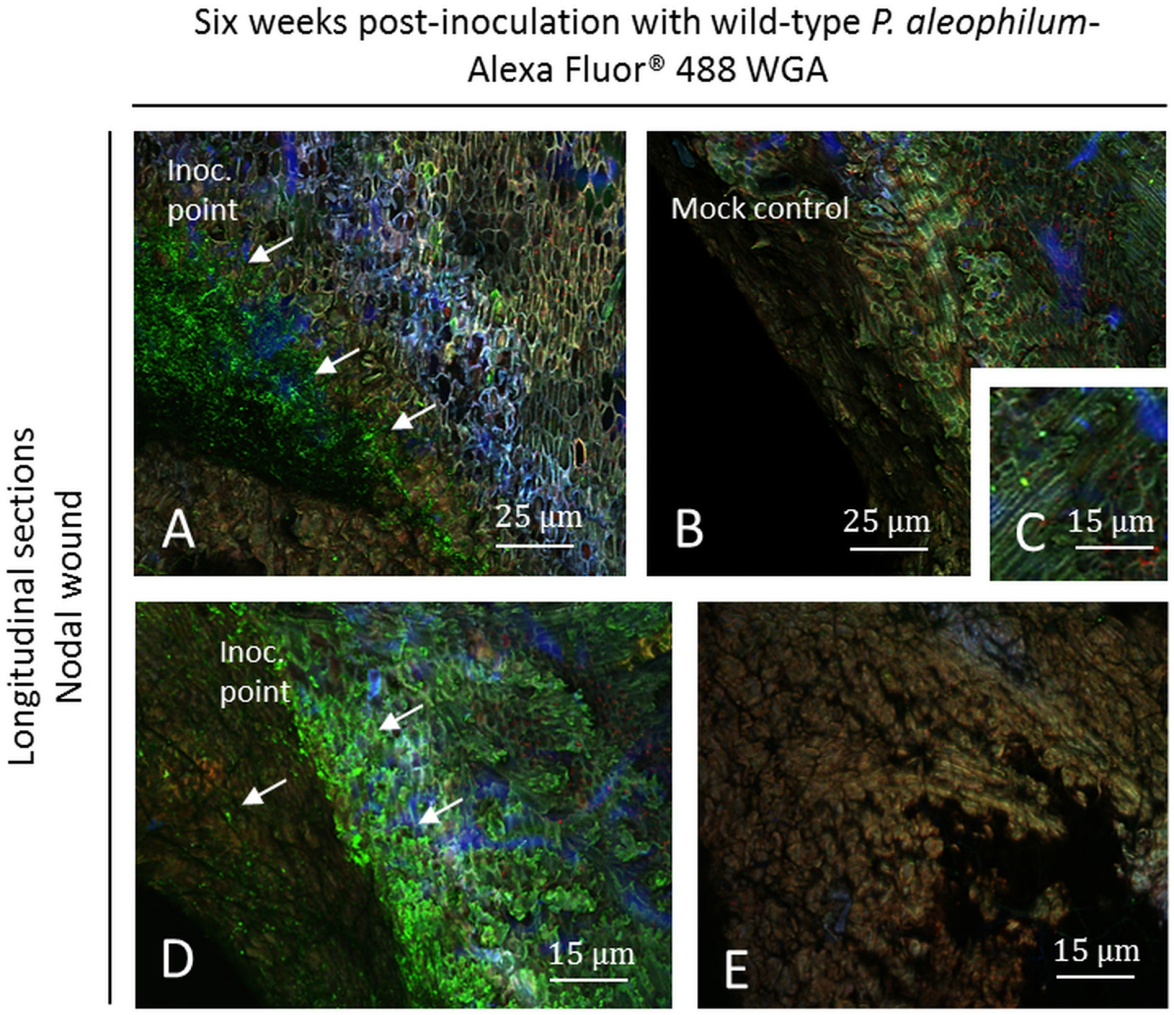
Observation of nodal transverse sections of Cabernet Sauvignon clone 15 cuttings challenged with wild-type *P*. *aleophilum* CBS 100398 (arrows) six weeks post-inoculation and coloured with Alexa Fluor 488-WGA. A) Inoculation point showing *P*. *aleophilum* and a strong plant response, as indicated by blue, pink and green fluorescence. Sometimes this plant defence reaction was yellow fluorescent. B–C) Mock control. D) Magnification of the zone colonised by the fungus (C) or not (E). Inoc. point: inoculation point.

Six weeks post-inoculation at the node level, hyphae were detected at the point of inoculation where a plant response was detected near this zone (Fig 6A). As for samples inoculated with *P*. *aleophilum*::*gfp*7, a higher fluorescence of blue, green and pink cells was observed on samples inoculated with the wild-type strain of *P*. *aleophilum* (Fig 6A) than in the node of mock plants (Fig 6B). These zones did not cover all of the inoculation point, similar as observed with *P*. *aleophilum*::*gfp*7. Parts of the inoculation point were colonised (Fig 6D), while others were not (Fig 6E). No granules reported with samples of *P*. *aleophilum*::*gfp*7 were recorded. Few microbes were recorded in control plants, especially in fibres (Fig 6C).

### CSLM microscopy of *P*. *aleophilum*::*gfp*7 twelve weeks post-inoculation

**Fig 7:**
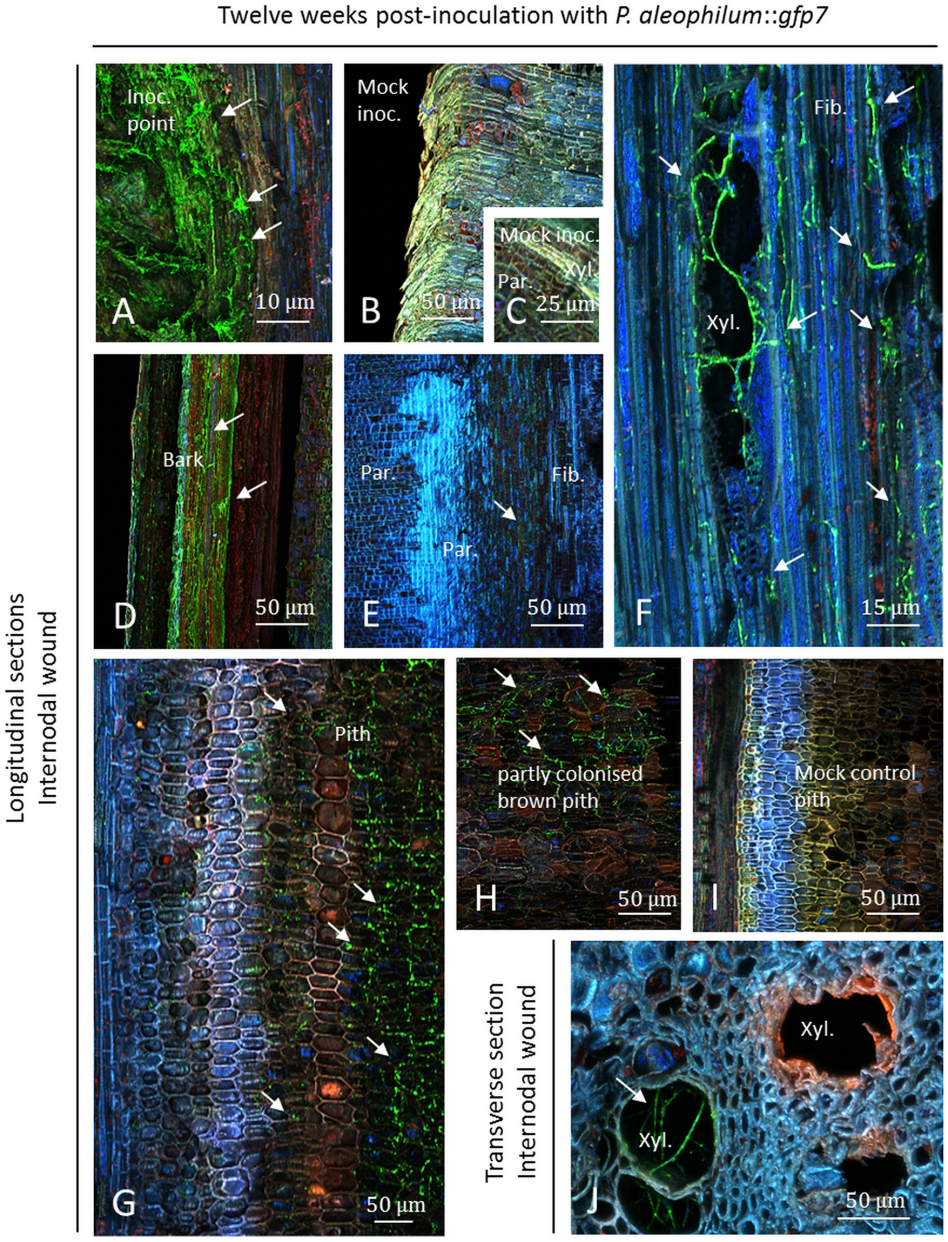
Observation of internodal longitudinal (A–H) and transverse (I) sections of Cabernet Sauvignon clone 15 cuttings challenged with *P*. *aleophilum*::*gfp*7 (arrows) twelve weeks post-inoculation. A) Inoculation point colonised by hyphae of *P*. *aleophilum*::*gfp*7. B–C) Mock control. D) Damaged bark covered with hyphae of *P*. *aleophilum*::*gfp*7. E) Fibres colonised by short hyphae of *P*. *aleophilum*::*gfp*7, with a blue autofluorescent reaction in parenchymal cell tissues next to them. F) Xylem vessel lumen strongly colonised by *P*. *aleophilum*::*gfp*7 with hyphae longer than in fibres. G–H) Brown pith colonised by *P*. *aleophilum*::*gfp*7 hyphae with fluorescent reaction zone not colonised (see cells not colonised in G). I) Pith control. J) *P*. *aleophilum*::*gfp*7 in metaxylem and some xylem elements not colonised but with orange/red fluorescence (a strong yellow fluorescence was also recorded). Fib.: fibres, Inoc. point: inoculation point, Mock inoc.: mock inoculated, Par.: parenchyma, Xyl.: xylem.

At the internode level, the inoculation point was densely colonised with long hyphae (Fig 7A) in comparison to mock controls where no hyphae were detected (Fig 7B and 7C). Hyphae were also visualised in the bark (Fig 7D) and short hyphae were observed in fibres where a strong response was observed in parenchymal cells near the fibres (Fig 7E) in all the samples but not along the entire fibre zone. Fibres were colonised by short hyphae while in the xylem lumen longer hyphae were frequently observed (Fig 7F). Hyphae passing from fibre cell layers to the xylem lumen were additionally visualised in the ones colonised (Fig 7F). In the pith and xylem fibres, long hyphae were visualised with a colonisation zone up to 8 mm up or down from the point of inoculation and this was not seen for xylem elements. The pith appeared as destroyed and brown in comparison to the control treatment (Fig 7G–7I). Interestingly, some cells inside the pith were not colonised by the fungus (Fig 7G and 7H) and some showed different fluorescence to the ones colonised by the fungus (Fig 7G and 7H). These cells were blue, pink or red fluorescent. Inside transversal sections the xylem lumen was colonised (Fig 7J). Destroyed xylem vessels were not colonised by the fungus and an orange/red fluorescence was recorded in these xylem vessels as described before (Fig 7J).

**Fig 8:**
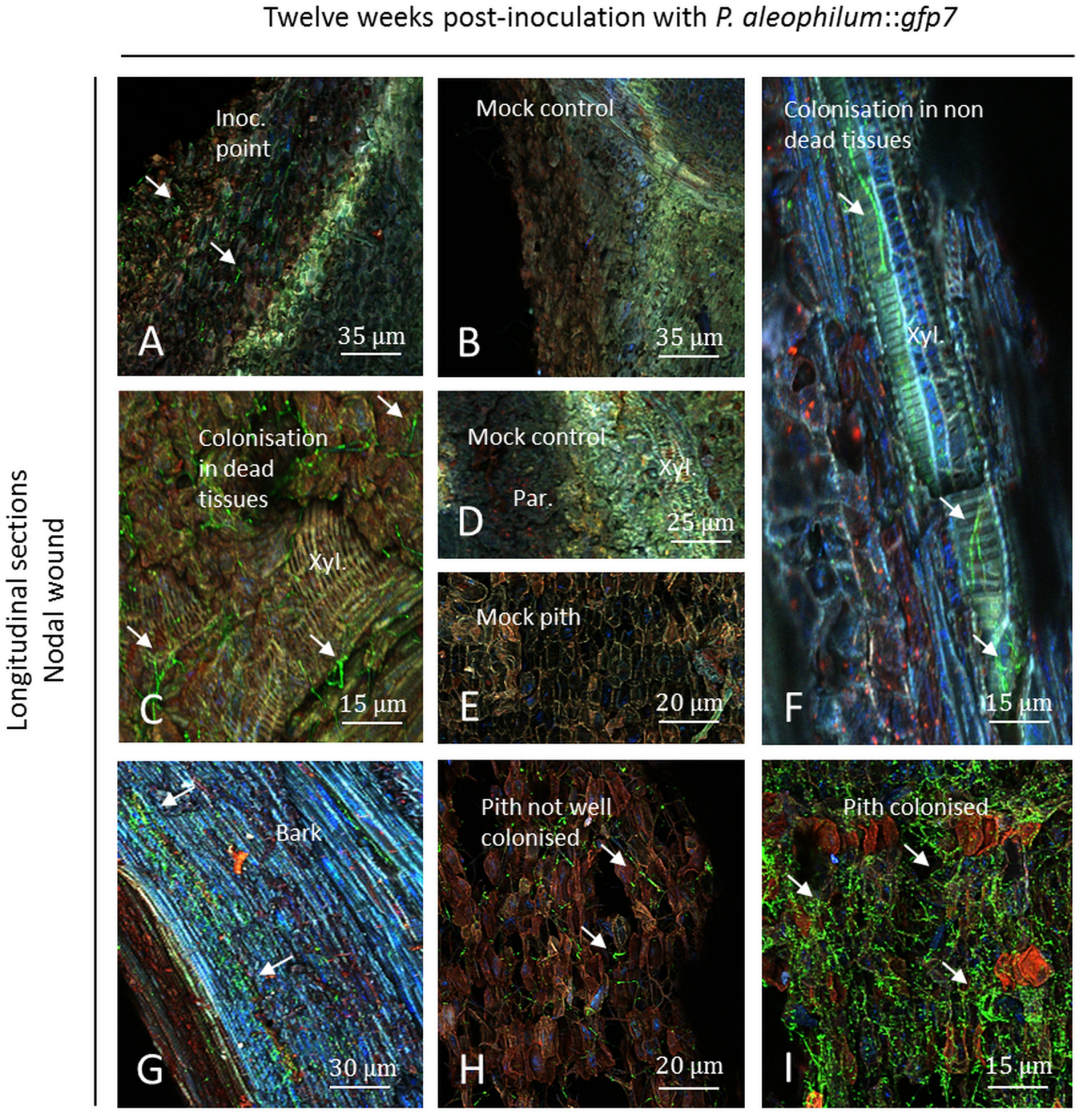
Observation of nodal transverse sections of Cabernet Sauvignon clone 15 cuttings challenged with *P*. *aleophilum*::*gfp7* (arrows) twelve weeks post-inoculation. A) Inoculation point showing a very low amount of *P*. *aleophilum*::*gfp*7 (arrows) and a strong plant response, appearing yellow and brighter than in control plants (B). C) Colonisation of dead tissues by *P*. *aleophilum*::*gfp*7 in fibres and xylem elements. D–E) Parenchymal cells, xylem, and pith from mock controls. F) Hyphae of *P*. *aleophilum*::*gfp*7 in a xylem vessel. G) Bark colonised by *P*. *aleophilum*::*gfp*7. H–I) Brown pith sparsely or densely colonised. Inoc. point: inoculation point, Par.: parenchyma, Xyl.: xylem.

At the node level, and at the point of inoculation, plant responses and a necrotic zone were observed for plants inoculated with *P*. *aleophilum*::*gfp*7 (Fig 8A). Control plants presented similar responses adjacent to a necrotic zone (Fig 8B and 8D). Less colonisation was recorded in comparison to the internodal inoculation twelve weeks post-inoculation. A colonisation of fibres and xylem elements was observed in dead tissues (Fig 8C). Hyphae were also observed inside the lumen of xylem of living plant parts, but always near the point of inoculation (Fig 8F). The bark was found to be colonised by short hyphae (Fig 8G). In the pith, some parts were detected as brown and not well colonised or alternatively densely colonised (Fig 8H and 8I). A brown pith was also found in some cases in control plants (Fig 8E) but more brownish tissues were recorded each time in case of *P*. *aleophilum*::*gfp*7 inoculation. This pith was colonised 8 mm above and below the point of inoculation.

### CSLM visualisation of wild-type *P*. *aleophilum* CBS 100398 twelve weeks post-inoculation using Alexa Fluor 488-WGA

**Fig 9:**
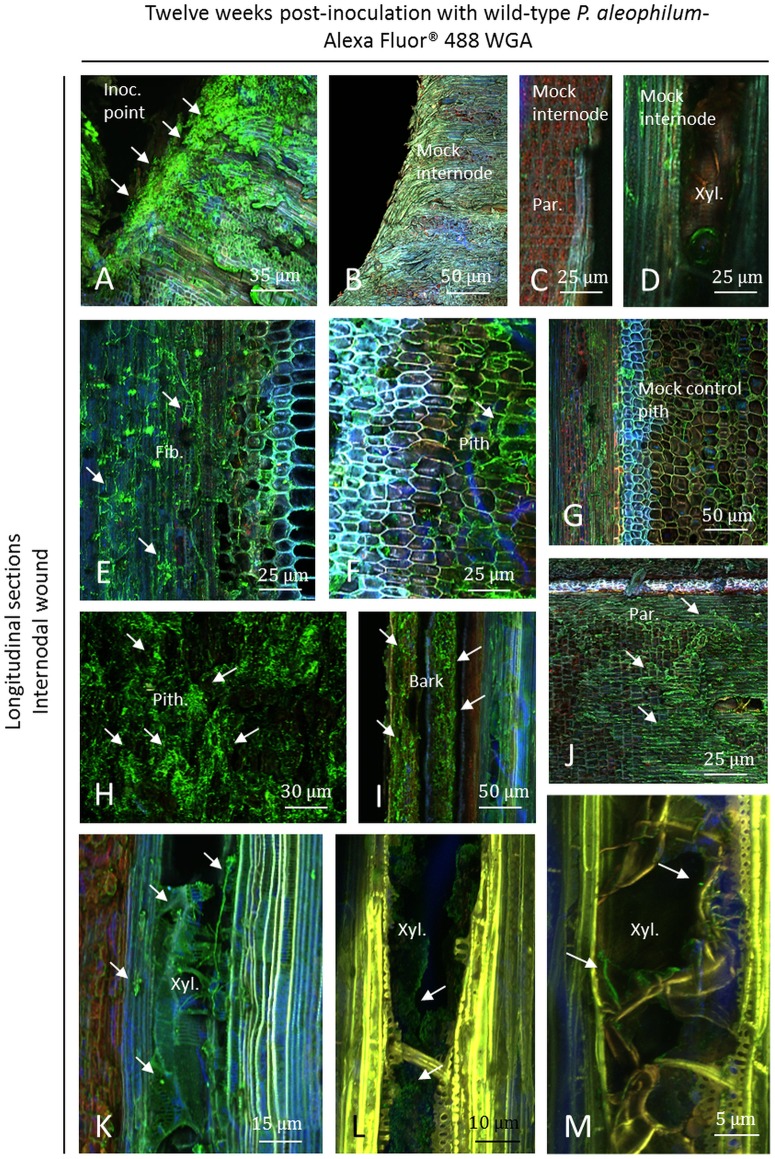
Observation of internodal longitudinal sections of Cabernet Sauvignon clone 15 cuttings challenged with wild-type *P*. *aleophilum* CBS 100398 (arrows) twelve weeks post-inoculation and stained with Alexa Fluor 488-WGA. A) Inoculation point colonised by *P*. *aleophilum*. B) Mock control. C) Parenchymal cells from a mock control. D) Xylem and fibres from a mock control containing endophytic microbes reacting to Alexa Fluor 488-WGA. E) Fibres colonised by *P*. *aleophilum*. F) Brown pith colonised by *P*. *aleophilum*. Some pith cells were not colonised but exhibited strong fluorescence. G) Control pith containing few hyphae of endophytic nature as well as in fibres and in the xylem zone. H) Brown/dark pith strongly colonised by *P*. *aleophilum*. I) Bark colonised. J) Presence of fungi in parenchymal cells surrounding a necrosis point. K-M) Hyphae or other microbial structures in xylem vessels, where tyloses have been also detected, with differences of fluorescence of the cell-walls (note the presence of microbial structures not corresponding to hyphae in L). Fib.: fibres, Inoc. point: inoculation point, Par.: parenchyma, Xyl.: xylem.

At the internode level, the inoculation point was densely colonised with long hyphae (Fig 9A) as seen in samples with *P*. *aleophilum*::*gfp*7 in contrast to control plants where no green fluorescent cells were observed (Fig 9B and 9C), except a few in fibres and xylem (Fig 9D). Fibres were heavily colonised in inoculated plants (Fig 9E). Hyphae were detected in the pith, which appeared brown, although some plant cells were not colonised (Fig 9F). Control piths were less brown but also contained few microbes (hyphae and bacteria of endophytic nature) (Fig 9G) but much less than inoculated plants. In samples inoculated with the wild-type strain of *P*. *aleophilum*, pith parts were strongly colonised and appeared in brown/dark colour with hyphae longer than in other tissues (Fig 9H). In tissues of the bark, hyphae were also visualised (Fig 9I). In the parenchyma, and near the inoculation point, a necrosis as brownish tissues was visualised and the presence of fungi was observed in parenchymal cells (Fig 9J) while no microbes and no necrosis were detected in control plants (Fig 9C). As for *P*. *aleophilum*::*gfp*7, pith and fibres were colonised up to 8 mm from the point of inoculation, whereas colonisation of xylem elements lagged behind. In most of the xylem vessels, a plant response was observed (Fig 9K–9M). Tyloses were frequently seen and cell-walls of the xylem elements showed yellow or sometimes orange fluorescence (Fig 9I and 9J). In these xylem elements, hyphae or structures from other microbes were observed (Fig 9K–9M), but not in every xylem element. Xylem vessels in mock-treated plants also presented few tyloses (Fig 9D).

**Fig 10:**
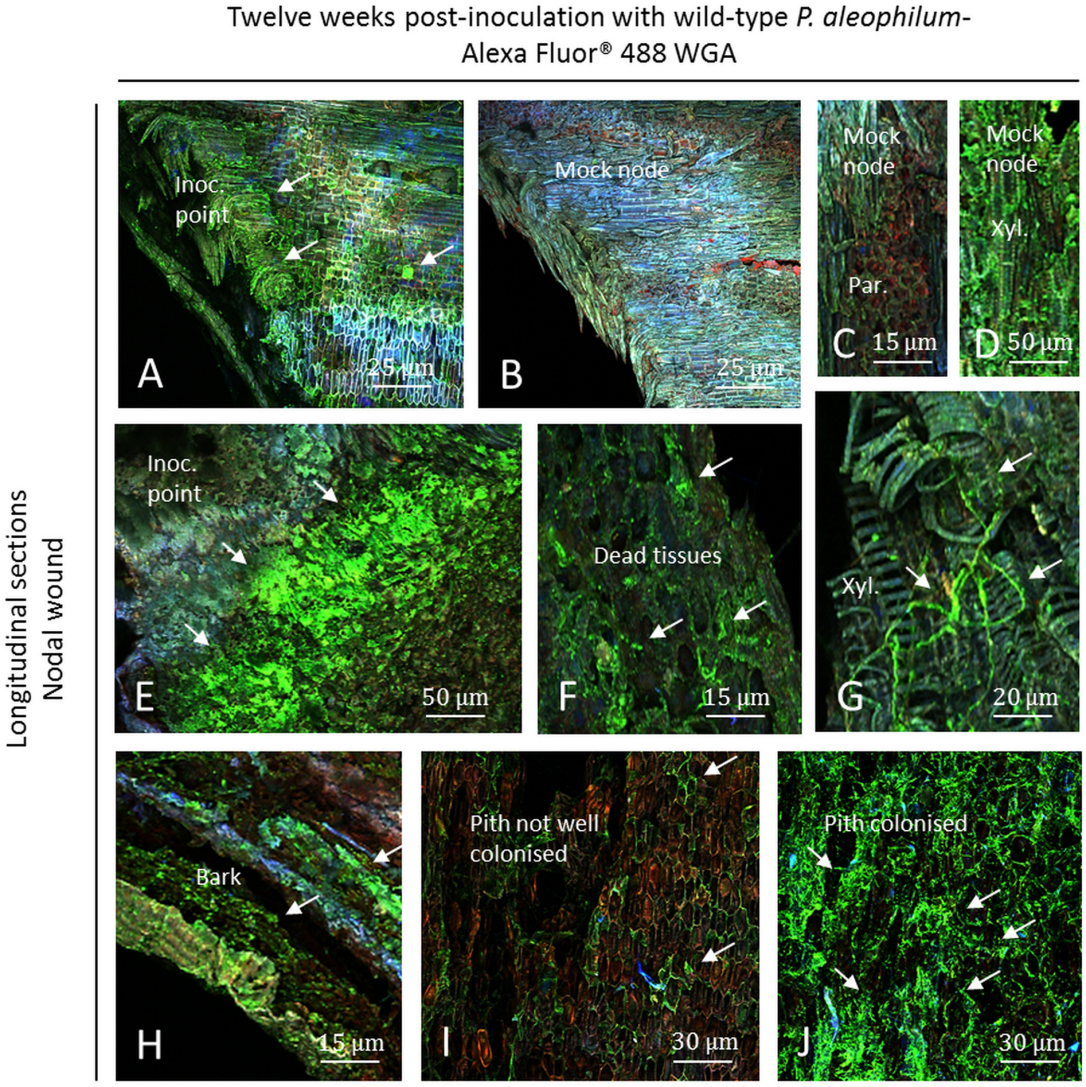
Observation of nodal transverse sections of Cabernet Sauvignon clone 15 cuttings challenged with wild-type *P*. *aleophilum* CBS 100398 (arrows) twelve weeks post-inoculation and coloured with Alexa Fluor 488-WGA. A) Inoculation point showing a very low amount of *P*. *aleophilum* (arrows) and a strong plant response, brighter than in control plants (B) and yellow. C) Parenchymal cells from a mock control. D) Xylem and fibres from mock controls containing endophytic microbes reacting to Alexa Fluor 488-WGA. E) Close view of the inoculation point showing colonisation of green fluorescent fungi. F–G) Colonisation of dead tissues by hyphae in parenchymal cells, fibres and xylem elements. H) Bark colonised by *P*. *aleophilum*. I–J) Brown/dark pith sparsely or densely colonised. Inoc. point: inoculation point, Par.: parenchyma, Xyl.: xylem.

At the node level, plant responses were less intense twelve weeks post-inoculation than six weeks post-inoculation (Fig 10A). The plant responded similarly to *P*. *aleophilum* CBS 100398 treatment and mock treatment twelve weeks post-inoculation (Fig 10A and 10B). Hyphae were observed at the node level (Fig 10E) with less colonisation than at the internode. Some dead tissues were observed as containing hyphae (Fig 10F) and xylem elements were colonised by hyphae (Fig 10G). Fewer amounts of microbes were visualised in control plants and nothing was detected in some tissues (Fig 10B–10D). In inoculated plants, green fluorescent hyphae were also detected as shorter hyphae in the bark near the point of inoculation (Fig 10H). The pith was brown/dark and either sparsely (Fig 10I) or heavily colonised (Fig 10J). Few hyphae and bacteria were recorded in control plants using Alexa Fluor 488-WGA.

## Discussion

*P*. *aleophilum* is considered as a pioneer in esca pathology. Understanding early events of trunk colonisation by these agents requires biomarkers to specifically localise a microorganism in its ecological niche. *gfp*-modified organisms are routinely used under laboratory conditions [22,26,29]. In this study, we analysed the behaviour of one strain of *P*. *aleophilum* marked with the *gfp* gene and further compared it to a wild-type strain of *P*. *aleophilum* using an Alexa Fluor 488-WGA probe and mock-treated plants. This probe preferentially binds to the chitin of all fungal species, and thus does not only report the presence of *P*. *aleophilum* specifically, but was also required to compare the wild-type strain and *gfp* marked strain *in planta*. On plants inoculated with *P*. *aleophilum* or control plants stained with Alexa Fluor 488-WGA, we detected natural endophytes. This is not surprising as cuttings were not sterile and it is well known that grapevine plants host natural endophytic microbes such as bacteria and fungi (natural microbes described in cuttings of [30]; and microflora inside twigs from the field [28,31]. WGA can detect fungi as well as gram-positive bacteria, archaea and other microbes (protozoa) as this lectin binds to N-acetyl-D-glucosamine and sialic acid [32–34]. Cuttings were treated, however, with a fungicide and with ethanol before use and the soil was sterilised. Less endophytes were seen in cuttings in comparison to what is known with twigs in the field (see for instance pictures and supplementary information in [28]). Indeed, only a small amount of natural endophytes were detected. This highlights however the need of a *gfp* transformant to study the colonisation of grapevine by *P*. *aleophilum*. This low amount of endophytic microflora also suggests that most of the Alexa Fluor 488-WGA signal observed in plant inoculated with the wild-type strain reported the presence of *P*. *aleophilum*. Moreover, colonisations of *P*. *aleophilum*::*gfp*7 and of wild-type *P*. *aleophilum* were similar in all different modalities tested: six weeks or twelve weeks post-inoculation, in the internode or the node of grapevine cuttings. For this reason, the colonisation of *P*. *aleophilum* is discussed without distinction of the transformed or wild-type strains. The only exception is that in some plants at 6 weeks post-inoculation we detected some ovoid granules in the case of *P*. *aleophilum*::*gfp*7 but not with the use of wild-type *P*. *aleophilum*. It remains speculative to discuss to what these materials can be, and it may be correlated to natural endophytes in some specific plants, as material was harvested from the vineyard and only few plants showed this response.

This study compares the early colonisation behaviour of *P*. *aleophilum* in two different plant tissues six and twelve weeks post-inoculation. Cuttings from Cabernet Sauvignon clone 15 were inoculated at the internodal and nodal regions and differences were recorded regarding fungal colonisation. The inoculation was efficient because the wounded region was covered with hyphae in comparison to control plants six and twelve weeks post-inoculation, for both internodal and nodal inoculation. In addition, symptoms were observed in plants inoculated with both strains, transformed and wild-type, twelve weeks post-inoculation as the wood of inoculated plants presented brownish/dark discolorations. The inoculation of a plug of hyphae is probably not comparable to natural conditions, but was necessary for this fundamental model designed for the understanding of early colonisation events by *P*. *aleophilum* in the trunk of young cuttings of Cabernet Sauvignon, and this pathosystem allows us to observe microscopic symptoms. *P*. *aleophilum* infection from plant to plant could occur in the vineyards because of conidia dispersal [9]. Nevertheless, infection of healthy wood may also originate from the bark next to the injury. Indeed, *P*. *aleophilum* can survive 12 weeks on the bark and still remain active under laboratory conditions. Consequently, both conidia and hyphae inocula should be used in a reductionist pathosystem designed to study wood diseases.

In this study samples were not fixed to avoid a loss of GFP signal. During sections, hyphae or conidia in some zones might have moved slightly. However, we did not see any not attached hyphae or conidia to the tissues at the microscopic scale when analysing the tissues, suggesting that this might be minor regarding the whole process. Microscopy was moreover performed in the whole samples to determine the niches of the fungus. Moreover, Z scans using confocal microscope were done to go partially in-depth into the tissues to avoid any contaminant. This was necessary to understand niches of *P*. *aleophilum* inside grapevine plants.

Early colonisation of grapevine trunk tissues (six and twelve weeks post-inoculation) seems to be more successful when inoculating *P*. *aleophilum* in the internode than in the node after cutting off a branch. In the internode, the fungus colonised, six weeks post-inoculation, the inoculation point and is limited to xylem fibres, pith and the bark, with a stronger colonisation of fibres compared to other tissues. The parenchyma was also colonised but to a smaller extent than the xylem fibres, and very few numbers of hyphae could be observed inside xylem vessels. At the internode level, the pith (appearing as brown or dark) and xylem fibres were strongly colonised twelve weeks post-inoculation by *P*. *aleophilum*. The bark was also colonised. Colonisation of the lumen of xylem vessels was additionally detected but the presence of tyloses blocked the progression of the fungus. The formation of tyloses has also been observed in mock-treated plants. This is not surprising because vessel obstruction, involved in the compartmentalisation of wood decays, cannot be separated from the plant response to wounding damage in our model [35–37]. Interestingly, the fungus spreads up to 8 mm from the inoculation site along the trunk. In the upper wood section, *P*. *aleophilum* was found in the pith, the parenchyma and xylem fibres as well as inside the bark but only a low signal was detected in xylem vessels in comparison to the point of inoculation. The location of *P*. *aleophilum* inoculated in young cuttings is similar to observations of the niches revealed using electron microscope made by Valtaud *et al*. [17] and with a non-*gfp* transformant. An inoculation of single-bud *in-vitro* plants growing aseptically was shown to result in the colonisation of all tissues forming the stem or roots and even leaves, a fact that is not considered possible under field conditions [15]. Using a FITC-WGA assay, Fleurat-Lessard *et al*. [18] visualised the fungus one year after inoculation in several parts of the trunk of the infected cuttings of cv. Ugni blanc, mainly inside xylem vessels and fibres, but also in proto-xylems, pith and rays. Comparatively, using an FITC-labelled serological approach they presented a similar localisation of the hyphae in the lumen of xylem vessels, vessel-associated cells, fibres and rays. Remarkably Fleurat-Lessard *et al*. [18] showed that only cell-walls of xylem fibres were damaged by *P*. *aleophilum*. The wood fibres seem thus to be an important tissue for the early events in *P*. *aleophilum*-grapevine interaction. Less active wood fibres, next to xylem vessels, may constitute a lignified shelter for *P*. *aleophilum* although the fungus can subsequently reach the xylem lumen.

At the node level, the fungus was restricted at the point of inoculation six weeks post-inoculation. This tissue responded strongly to treatments, nevertheless *P*. *aleophilum* was detected in the bark, dead tissues as well as inside the pith, xylem fibres and xylem vessels twelve weeks post-inoculation. The progression of the fungus inside plants was recorded up to 8 mm from the point of infection twelve weeks post-inoculation. Symptoms of wood decay were also recorded.

Different plant responses were recorded during progression of the fungus. A grapevine wood response was observed in both nodal and internodal tissues. Plant responses in the node were higher than in the internode. In the node, and especially in transversal sections, an intense reaction zone was visible six weeks post-inoculation in plants inoculated with *P*. *aleophilum* and mock-treated plants. This response can be considered as two components: a wood response to wounding observed in mock treatments, and a wood response to *P*. *aleophilum*. Indeed, *P*. *aleophilum* induced a blue, green and pink fluorescence more intense than in mock-treated plants six weeks post-inoculation. This difference of wood response in the node disappeared twelve weeks post-inoculation. The reaction zone was similar in inoculated plants and mock-treated plants. Consequently, the grapevine response in the node varied according to the time post-treatment and it seems correlated to the presence of *P*. *aleophilum* six weeks post-inoculation and only related to wound healing twelve weeks after inoculation.

Plant responses in the internode were not visible six weeks post-inoculation. Twelve weeks post-inoculation a fluorescent plant response was visualised in different tissues such as pith, parenchymal cells near fibres and in xylem vessels. Interestingly, those fluorescent cells were not colonised by the fungus or rarely colonised by only a small number of hyphae. This suggests that plant responses may prevent the colonisation of some cells by *P*. *aleophilum* without hampering a colonisation of the plant up to 8 mm from the inoculation point twelve weeks post-inoculation. We observed that the fungus reaches xylem vessels following fibre colonisation, and progresses slowly inside plants using fibres and pith that are degraded. Xylem colonisation is blocked by tylose formation. Defence reactions following inoculation of *P*. *aleophilum* have been well documented previously (see [38,39]) and correspond to what we have seen in this study, except that we observed plant responses under different wavelength of fluorescence.

The response of trunk tissues to fungal pathogens is considered to be general and nonspecific [40,41], but this question has to be addressed molecularly to reveal whether the perception exhibits specificity or not. Our study suggests for the first time that trunk tissues may respond particularly to an esca-associated agent six weeks post-inoculation in the node. These events seem to occur quite early because plant responses to wounding and to the presence of *P*. *aleophilum* were similar twelve weeks post-inoculation.

In this work, we focused on monitoring the colonisation by *P*. *aleophilum* and revealed—by using both a *gfp* mutant as well as its wild-type—that *P*. *aleophilum* colonises more efficiently when entering the plant at the internode than at the node. However, so far *gfp* marked strains have not been applied for studying the colonisation behaviour of *P*. *aleophilum* specifically. The use of a *gfp* marked strain has been done with another esca-associated fungus *P*. *chlamydospora* [24] using *Vitis vinifera* L. ‘Montepulciano’, ‘Verdicchio’, ‘Sangiovese’, ‘Biancame’, and ‘Cabernet Sauvignon’; and the grapevine rootstocks ‘Kober 5BB’, ‘SO4’, ‘420A’, ‘1103P’, and *V*. *rupestris*. The expression of the *Pch*-sGFP71 transformed line was localised in the xylem area, primarily around vessels. The use of a *DsRed*-labelled *P*. *chlamydospora* has also confirmed a preference for the xylem compared to the pith of Cabernet-Sauvignon and Sauvignon Blanc cuttings [23]. However, no report of a *gfp* transformed *P*. *aleophilum* and its study of colonisation has been previously done.

In our study, we showed that the higher colonisation of the internode was linked to the colonisation of xylem fibres prior to the later colonisation of xylem vessels and that the bark and pith contribute to the progression of the fungus. Interestingly, the node that was less colonised compared to the internode was the tissue responding the most to treatments. Grapevine wood responds to wounding and may also provide a particular response to *P*. *aleophilum*. The nature of these responses and its capacity to protect the plant will be an important focus for future studies. A better understanding of esca-associated fungal colonisation in grapevine wood tissues will be gained through the full appreciation and elucidation of the complex pathosystem, which is essential in order to develop alternative strategies to control the disease.
